# Personal

**Published:** 1919-06

**Authors:** 


### PERSONAL.

Dr. J. Spencer David has been promoted to the rank of captain and has received his discharge from the United States Army to return to the practice in Dallas.

Mrs. Margaret Sullivan, an army nurse who was gassed while attending patients in the Argonne fighting and who underwent the same hardships that the best of soldiers endured, is back at her home at Alma, Texas.

Dr. J. A. Wolfe after an absence of 21 months serving his country in France has associated himself with the firm of Gumby, Hoard, McIlhanon and Spangler of Sherman in the practice of medicine.

Dr. T. H. Harrell, who has returned to his home at Gonzales, was promoted from the rank of Major in the Medical Corps to the rank of Lieutenant Colonel before being discharged.

Dr. Frank G. Sanders, after an absence of 18 months, is located at the Johnson-Beall Sanitarium in Fort Worth.

Dr. John O. McReynolds of Dallas, who has had the rank of Major, has been commissioned a Colonel in the Officers Reserve Corps of the United States Army.

Captain Leslie C. Frank, who has just received his discharge from the Army service, has been appointed Director of Health for Dallas.

Dr. F. F. Jones, one of the best known practicing physicians of Sherman, died on June 12th as a result of injuries sustained when his automobile was struck by a train. Dr. Jones was a member of the class of 1899 from the Medical School of the State University.

Dr. Leslie Moore has been elected president of the North Texas Medical Association for the 18th consecutive time. Dr. D. J. Wilson was elected vice-president, Dr. David L. Bettison, secretary, Dr. M. L. Wilbanks, treasurer, Dr. A. B. Small, councilor. The next meeting will be in Fort Worth in December.

Dr. R. M. Prather has his discharge from the Reserve Medical Corps of the United States Army and will return to Beeville to resume the practice of law.

Dr. J. T. Carter of Rice, Texas, is improving after an operation at Corsicana for appendicitis.

When Dr. R. B. Warren of Zavalla, Texas, left his home for a month's stay at Marlin to recuperate from excessive work, he left that town temporarily without a physician for the first time in its history.

Dr. J. W. Orion of Ft. Worth, has recovered from an operation for appendicitis.

Dr. W. M. Trimble, county health officer of Fort Worth, has warned the physicians of that county that if required reports of all communicable diseases are not made, criminal prosecution will be instituted.

Dr. J. T. Field, retired physician of Fort Worth, and Miss Mary Jordan, of that city, were married recently. They are spending the summer at Dr. Field's ranch near Rockport.

Dr. R. L. Harris, of Cleburne, has purchased the Southwestern Sanitarium in the suburbs of Cleburne from T. C. Jackson.

Dr. J. M. Armstrong has resumed his practice at Garland, Texas, after a time spent in recuperating his health at Kerrville.

Captain G. C. Kinley, of the United States Medical Reserve Corps, has been discharged and will resume his professional work in Dallas.

